# Regulated Extracellular Choline Acetyltransferase Activity— The Plausible Missing Link of the Distant Action of Acetylcholine in the Cholinergic Anti-Inflammatory Pathway

**DOI:** 10.1371/journal.pone.0065936

**Published:** 2013-06-19

**Authors:** Swetha Vijayaraghavan, Azadeh Karami, Shahin Aeinehband, Homira Behbahani, Alf Grandien, Bo Nilsson, Kristina N. Ekdahl, Rickard P. F. Lindblom, Fredrik Piehl, Taher Darreh-Shori

**Affiliations:** 1 Division of Alzheimer Neurobiology Center, Karolinska Institutet, Department of Neurobiology, Care Sciences and Society, Huddinge, Stockholm, Sweden; 2 Division of Alzheimer Disease Research Center, Karolinska Institutet, Department of Neurobiology, Care Sciences and Society, Huddinge, Stockholm, Sweden; 3 Department of Medicine, Center for Hematology and Regenerative Medicine, Huddinge, Stockholm, Sweden; 4 Department of Clinical Neuroscience, Unit for Neuroimmunology, Solna, Stockholm, Sweden; 5 Department of Immunology, Genetics and Pathology, Division of Clinical Immunology, Uppsala University, Uppsala, Sweden; 6 Linnæus Center of Biomaterials Chemistry, Linnæus University, Kalmar, Sweden; Weizmann Institute of Science, Israel

## Abstract

Acetylcholine (ACh), the classical neurotransmitter, also affects a variety of nonexcitable cells, such as endothelia, microglia, astrocytes and lymphocytes in both the nervous system and secondary lymphoid organs. Most of these cells are very distant from cholinergic synapses. The action of ACh on these distant cells is unlikely to occur through diffusion, given that ACh is very short-lived in the presence of acetylcholinesterase (AChE) and butyrylcholinesterase (BuChE), two extremely efficient ACh-degrading enzymes abundantly present in extracellular fluids. In this study, we show compelling evidence for presence of a high concentration and activity of the ACh-synthesizing enzyme, choline-acetyltransferase (ChAT) in human cerebrospinal fluid (CSF) and plasma. We show that ChAT levels are physiologically balanced to the levels of its counteracting enzymes, AChE and BuChE in the human plasma and CSF. Equilibrium analyses show that soluble ChAT maintains a steady-state ACh level in the presence of physiological levels of fully active ACh-degrading enzymes. We show that ChAT is secreted by cultured human-brain astrocytes, and that activated spleen lymphocytes release ChAT itself rather than ACh. We further report differential CSF levels of ChAT in relation to Alzheimer’s disease risk genotypes, as well as in patients with multiple sclerosis, a chronic neuroinflammatory disease, compared to controls. Interestingly, soluble CSF ChAT levels show strong correlation with soluble complement factor levels, supporting a role in inflammatory regulation. This study provides a plausible explanation for the long-distance action of ACh through continuous renewal of ACh in extracellular fluids by the soluble ChAT and thereby maintenance of steady-state equilibrium between hydrolysis and synthesis of this ubiquitous cholinergic signal substance in the brain and peripheral compartments. These findings may have important implications for the role of cholinergic signaling in states of inflammation in general and in neurodegenerative disease, such as Alzheimer’s disease and multiple sclerosis in particular.

## Introduction

Inflammatory processes are involved in the pathogenesis of a variety of degenerative diseases, such as Alzheimer’s disease (AD), multiple sclerosis (MS) and rheumatoid arthritis (RA). More recent studies have established that acetylcholine (ACh), the classical neurotransmitter in the central and peripheral nervous systems, acts as a suppressor of inflammatory responses of lymphocytes, mediated by binding to α7-nicotinic ACh receptors (α7-nAChRs) [1]. This is known as the cholinergic anti-inflammatory pathway, by which the nervous system is proposed to exert immunomodulatory effects on systemic immunity [2], [3].

However, there are still unresolved questions regarding this hypothesis. In particular that (i) ACh must be able to diffuse considerable distances from the cholinergic nerve terminals and (ii) resist the action of two extremely efficient ACh-degrading enzymes, acetyl- (AChE) and butyryl-cholinesterase (BuChE), which are abundant in extracellular fluids such as plasma and cerebrospinal fluids (CSF). In addition, the immune-suppressive activity requires that ACh has to be present at certain extrasynaptic levels to exert its putative role on immune cells by way of activating α7-nAChR ion-channels.

Additional questions arise from our recent demonstration in patients with AD that beta-amyloid (Aβ) peptides, the main component of senile plaques in the AD brain, together with high ApoE protein interact physically with BuChE and AChE[4]–[6]. This leads to formation of highly stable and soluble BuChE/AChE-Aβ-ApoE complexes (BAβACs) in CSF[4]–[6]. In AD CSF, the BAβACs appear dormant but gain ultrafast ACh hydrolyzing activity with addition of Aβ peptides. This indicates that BAβACs can oscillate between a slow and ultrafast state of ACh hydrolysis and that Aβ acts as their “turn-on” switch [4], [6]. Thus, an Aβ-induced allosteric hyper-activation of these enzymes may represent a native function for universal production and nerve activity-synchronized Aβ release into synapses and interstitial fluid (ISF) [7]. In other words, the physiological action of Aβ can include the tuning of cholinergic action at both synapses and in ISF, thereby affecting the activity status of cholinoceptive neuronal and non-neuronal nonexcitable cells, which are abundant in the brain and include microglia, astrocytes, oligodendrocytes, endothelia, and vascular smooth muscles [8]–[13].

Thus, an abnormal formation and accumulation of BAβACs at synapses or within the brain parenchyma provides a plausible explanation for the main characteristic features of AD, namely the selective deficit in the cholinergic signaling [4], [6] and the presence of low-grade chronic inflammation, neuronal disconnection, regional cerebral blood flow, and metabolic disturbances. These are particularly seen in patients carrying the main genetic risk factor of non-familial AD, namely the ε4 allele of Apolipoprotein E (APOE4)[14]–[16].

However, it is difficult to conceive how hyperactivation of two enzymes with high intrinsic ACh-hydrolyzing capacity may have a meaningful pathophysiological impact on extrasynaptic ACh levels. In order to balance the actions of highly abundant and efficient cholinesterases, we hypothesized that ACh-synthesizing machinery is also present in extracellular fluids, whose function is to uphold steady-state equilibrium of ACh levels. If so, nerve activity dependent release of Aβ would result in a transitory ACh-hydrolytic burst through hyperactivation of BAβACs, with rapid lowering of ACh levels. The subsequent re-uptake of Aβ peptides would terminate this regulatory signaling so that ACh equilibrium can be re-established.

The main ACh-synthesizing enzyme is choline acetyltransferase (ChAT), which was discovered about 70 years ago [17]. Until now, ChAT has been considered exclusively a cytosolic enzyme, where it synthesizes ACh, to be stored in vesicles until release by exocytosis into the synaptic cleft, where it subsequently is degraded into choline by membrane-anchored AChE or BuChE.

Several splice variants of ChAT (R-, N- and M-type) are generated from the coding gene region at the “cholinergic gene locus”. In human, the M-type mRNA transcript may produce both a larger (82 kDa) and a smaller (69 kDa) molecular isoform [18]. The human R and N types of ChAT mRNA produce only the smaller isoform [18]. Another, simpler classification refer to a common type (cChAT) in CNS and a peripheral type (pChAT), which is preferentially expressed by peripheral neurons with an expected Mw of 50 kDa [19]. There are also evidence for another mRNA splice variant that produces a small 27 kDa ChAT protein, which appears to lack catalytic activity but may have a regulatory role on the activity of full-length ChAT [20].

Both the well-established cholinergic-antiinflammatory hypothesis and our model where extracellular hydrolyzing activity is balanced by capacity to regenerate ACh are challenged by lack of convincing evidence for extracellular ChAT, with several conflicting reports[21]–[30].

The current study was hence initiated as a proof-of-concept, with the main objective of a thorough investigation if ChAT protein and activity is present in extracellular fluids of the peripheral and CNS compartments. We used human plasma and CSF as surrogates of the extracellular fluids in these compartments. We also used mouse lymphocytes and human-brain primary astrocytes and human embryonic stem cells to investigate the probable source of ChAT in the extracellular fluids.

We here demonstrate conclusive evidence for the presence of high levels of functional ChAT in human plasma and CSF. We show that CSF ChAT maintains a steady state level of ACh in the presence of physiological levels of fully active cholinesterases. We also show that ChAT is actively secreted by different non-neuronal cells, and that the levels of ChAT in the extracellular fluids are physiologically balanced to the levels of the counteracting ACh-degrading enzymes. We show that ChAT level is elevated in patients with MS compared to controls, and this strongly correlates to CSF levels of the complement factors of innate immune system.

## Results

### Dot-blot Analysis Shows Presence of Readily Detectable ChAT Protein Levels in CSF

**Figure 1:**
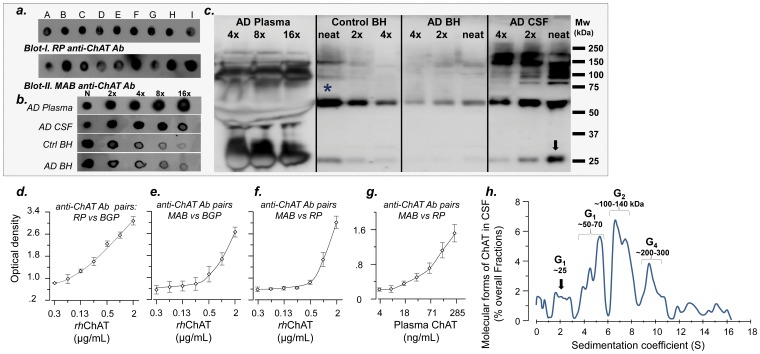
Detection and characterization of extracellular ChAT protein in human plasma and CSF. (a) Dot-blot analysis of nine different pooled CSF samples using two different antibodies. (b) The relative amount of ChAT in plasma or CSF, compared to brain homogenates (BH) of AD or control. At 16-fold dilutions, the immunosignals are much stronger in plasma and CSF compared to BH. This demonstrates the relative abundance of the ChAT protein in extracellular fluids. (c) Western-blot characterization of molecular form of ChAT in plasma, CSF and BH. The major detected protein band (*) in the BHs corresponds to a ChAT protein with a Mw of ∼65 kDa. In addition, there are several detected heavier molecular forms of ChAT in the CSF. All samples were loaded on one gel. Note also that the amount of the ∼65 kDa ChAT was so high in plasma that it distorted the gel downward. (d–g) Combinatorial sandwich ELISA results for identification of extracellular ChAT using three different antibody pairs for recombinant human ChAT (rhChAT), and a pooled plasma sample (g) calibrated for ChAT protein concentration using the rhChAT and ELISA setup in (f). (h) Further characterization of the molecular forms of CSF ChAT in consecutive fractions of pooled AD CSF samples separated by sucrose-density gradient technique, and the subsequent quantification of ChAT by sandwich ELISA. The graph represents the average of nine different pooled CSF. This independent analysis provides an identical pattern of molecular forms detected by Western analysis. Due to lack of prior reports, we used analogous terminology, which is used for the counteracting cholinergic enzymes, AChE and BuChE, that is, G_n_, where n denotes the number of globular subunits in each detected molecular form of ChAT in CSF. The molecular weights are calculated based on the known Mw of two internal standard proteins. In all dot-blot analyses, 2 µL of each sample (neat or diluted) was used. In the Western blot analysis, each lane was loaded with 15 µL of a mixture, containing 10 µL of sample (neat or diluted) and 5 µL of a 6x concentrated reducing Laemmli loading buffer. All ChAT protein quantifications were done with the ELISA antibody pair’s combination in (g). Anti-ChAT antibodies: RP = rabbit polyclonal antibody (Ab), BGP = biotin-labeled goat polyclonal Ab, MAB = mouse monoclonal Ab.

First, we used two selective antibodies against human ChAT and performed dot-blot analysis on pooled AD CSF and plasma samples (**Fig. 1a** **, Blots I** & **II**), and pooled brain homogenates (BH) from controls and AD patients (blots in **Fig. 1b**). We then investigated the relative amount of ChAT in plasma and CSF by serial dilution of the samples. A comparison of the relative signals of the immunostained dots (**Fig. 1b**), particularly at eight- to 16-fold dilutions, indicated that the amount of ChAT in plasma and CSF may be greater than that found in brain tissue. Thus, ChAT is not only present in CSF and plasma, but also at abundant concentrations compared to brain homogenates.

### Western-blot Analysis Confirmed Presence of High ChAT in CSF and Plasma

The findings obtained by dot-blot analyses were confirmed by Western blot (**Fig. 1c**). A comparison of the immunostained bands of the human brain homogenates and CSF and plasma (**Fig. 1c**) indicated that virtually all the different immunopositive bands in the brain homogenates were also present in CSF (and plasma) but at higher density. The strongest band in plasma, CSF, and brain homogenates had a molecular weight (Mw) of about 65 kDa (**Fig. 1c**). However, several additional clearly discernible bands were observed, particularly in CSF and plasma. These bands had Mw ranging between 80 kDa to >250 kDa. To the best of our knowledge, these analyses show for the first time that ChAT, like its counteracting enzymes AChE and BuChE, might exist as a variety of multimeric molecular forms in CSF and plasma.

### Sandwich ELISA Confirmed Presence of ChAT Protein in CSF and Plasma

As further proof of the identity of the detected protein bands, we designed a pair of sandwich ELISA assays using a combination of antibodies and purified recombinant human ChAT protein (**Fig. 1d–g**). The specificity of the sandwich ELISA is superior to Western and dot blotting, since it relies on detection of two different epitopes on the same protein, recognized by two independent antibodies. All the tested antibody combinations indicated that these antibodies indeed detected human ChAT (**Fig. 1d–g**).

### Sedimentation Analysis Confirmed that ChAT Exist as Multimeric Molecular Forms

Since the immunoblotting analysis indicated that numerous molecular forms existed in the samples, particularly in human plasma and CSF (**Fig. 1c**), we further characterized the molecular forms of ChAT protein in CSF by sucrose density gradient (SDG) analysis (**Fig. 1h**). This analysis strongly supported the immunoblotting observations and ensured that the immunostained bands in **Fig. 1c** were not artifacts from unspecific binding of antibodies. This is because we first separated the isoproteins of ChAT by ultracentrifugation and fractionation, and then determined the presence of ChAT in the fractions by highly selective sandwich ELISA assay.

As illustrated in **Fig. 1h**, ChAT is present as different molecular forms with distinct molecular weights. Since molecular forms of the counteracting cholinergic enzymes AChE and BuChE are named based on assembly of their globular subunits (G_n_), we here used an analogous terminology for the detected molecular forms of ChAT in CSF (**Fig. 1h**). The G_1_, G_2_, and G_4_ molecular forms of CSF ChAT had apparent Mw of 50–70 kDa, 100–140 kDa and 200–300 kDa, respectively (**Fig. 1h**). The dominant molecular form of ChAT (with respect to its concentration) in AD CSF was the G_2_ form. The SDG analyses also separated the much smaller band observed in the immunoblots (black arrow in **Fig. 1c & 1h**), which might represent the previously reported 27-kDa splice variant protein of ChAT [**20**].

### High Levels of ChAT Protein Exist in Human Extracellular Fluid, CSF and Plasma

**Figure 2:**
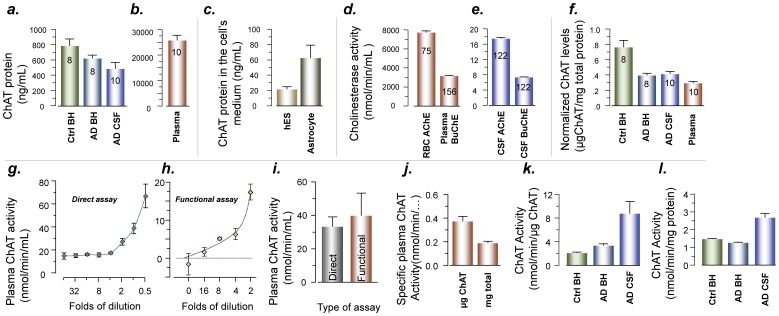
The extracellular ChAT protein is functionally intact and physiologically balanced in both plasma and CSF. (a–b) The quantity of the ChAT protein determined by ELISA in pooled brain homogenates of control brains (Ctrl BH) and AD brains (AD BH), AD CSF and plasma. The concentration of ChAT in the plasma is 50-fold higher (b) than the CSF samples (a). (c) The amount of ChAT in the cell-culture medium of human embryonic stem cells (hES) and human brain primary astrocytes, indicating that these cells readily released ChAT into the medium. (d–e) illustrate ∼ 300-fold higher overall activity of the counteracting enzymes, red blood-cell acetylcholinesterase (RBC-AChE), and plasma BuChE (d) compared to the AChE and BuChE activities in the CSF (e). These observations may suggest that ChAT levels in plasma and CSF (a–b) is balanced to the corresponding physiological levels of AChE and BuChE activities. (f) The amount of ChAT normalized to the total protein in the samples. (g–h) Two new, alternative, colorimetric assays of ChAT activity confirmed that the plasma and CSF ChAT are functionally intact. ChAT activity was measured directly (g) or after immunocapturing of the ChAT protein (h) into wells of microtiter plates coated with mouse monoclonal anti-ChAT antibody in a functional ELISA setup. (i) Comparison of measured ChAT activity by these two approaches, indicating that both approaches provide essentially similar levels of ChAT activity in pooled plasma samples. The functional assay also excludes any possible contribution or interference of other plasma constituents in the synthesis of acetylcholine (e.g., by carnitine acetyltransferase), since ChAT protein is first immunoadsorbed and the other plasma constituents are washed away before the substrates and other necessary reagents are added. (j–l) The plasma, CSF and brain ChAT activities normalized to quantified ChAT protein or the total protein in the samples. The digits in the column bar represent the number of samples that were analyzed.

We then quantified the amount of ChAT protein in nine different pooled CSF and plasma samples, as well as brain homogenates (**Fig. 2a–b**). This confirmed that ChAT protein was abundant in both plasma and CSF (**Fig. 2a, b & f**). This agrees with the dot- and Western-blot analyses.

### ChAT Protein Levels is Proportional to the Amount of Cholinesterases in CSF and Plasma

Since we found ChAT protein concentrations to be about 50-fold greater in plasma than in CSF (**Fig. 2b** vs **2a**), we also quantified the amount of ACh-degrading enzymes in the circulations as the sum of red blood cells (RBC-) AChE and plasma BuChE activities (**Fig. 2d**). This is because the relationship between these counteracting enzymes may be indicative of the biological equilibrium system to maintain certain levels of ACh in extracellular fluids.

The overall activity of cholinesterases (ChEs) in the peripheral circulation was 11 198±234 nmol/min/ml, whereas the corresponding value in CSF was just 25±1 nmol/min/ml (**Fig. 2d–e**). Thus, the relative amount of ChE activities was about 450-fold higher in peripheral circulation than in CSF. This indicates that the levels of ChAT found in plasma (**Fig. 2b**) and CSF (**Fig. 2a**) are reasonably proportional to the amount of ChE activity in the respective compartment. We also found that CSF and plasma levels of ChAT were similar when normalized for total protein content in the samples (**Fig. 2f**). These findings suggest that the presence of ChAT in extracellular compartments may fulfill a physiological function.

### CSF and Plasma ChAT is Functionally Intact and Highly Active

In order to test if ChAT protein present in CSF and plasma is functionally intact a simple sensitive colorimetric assay was applied. First, ChAT activity was directly measured in the samples using a microtiter plate (**Fig. 2g**). The second approach consists of immunocapturing ChAT molecules in the well of a microtiter ELISA plate and measuring the activity of immuno-adsorbed ChAT (**Fig. 2h**). We previously described a similar functional ELISA-like technique for measuring enzymatic activity of the related cholinergic degrading enzymes AChE and BuChE [14], [31]. Notably, this functional assay ensured that any observed activity is specifically generated by ChAT, as other constituents of the samples had been rigorously washed away through the preceding steps.

Both the functional ELISA assay and the overall direct assay yielded essentially similar levels of ChAT activity (**Fig. 2i**). In addition, these analyses demonstrated that plasma and CSF ChAT proteins were highly functionally active (**Fig. 2j–l**). Interestingly, a comparison between ChAT activity in plasma, CSF, and brain homogenate demonstrates that CSF has the highest activity normalized for total protein content in the samples (**Fig. 2j–l**). The lower relative activity of plasma might reflect greater proteolytic degradation or a nonlinear dose-response relationship to ChAT levels.

### ChAT Upholds an Extracellular ACh Equilibrium

**Figure 3:**
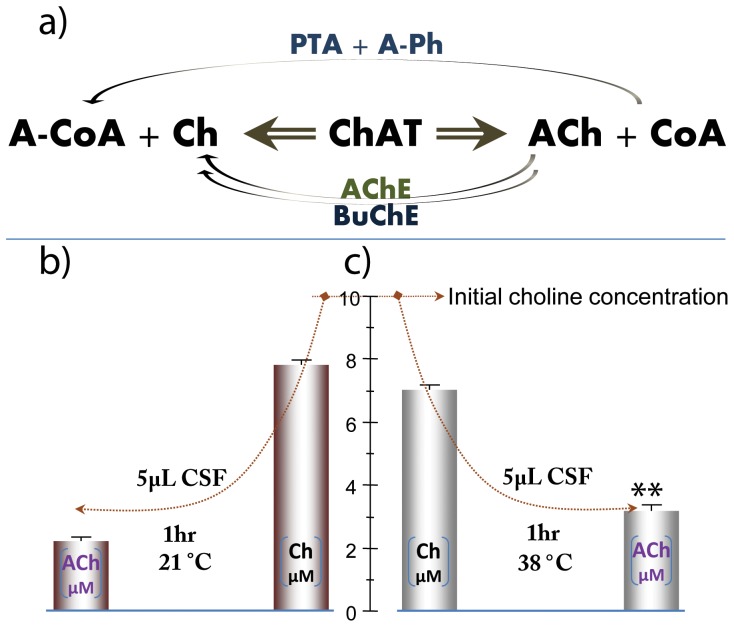
Extracellular CSF ChAT proteins maintain a steady-state level of ACh in the presence of naturally occurring levels of fully active cholinesterases. (a) Schematic illustration of the overall reaction setup. Pooled CSF sample was incubated for one hour with a cocktail containing 10 µM choline chloride and 50 µM acetyl-Coenzyme A (A-CoA). To avoid depletion of A-CoA, the accessory enzyme phosphotransacetylase (PTA) and acetyl-phosphate (A-Ph) were included in the cocktail to continuously regenerate the cofactor, A-CoA. (b) CSF ChAT maintains a steady-state ACh concentration of 2.11±0.27 µM after one hour at room temperature. However, ChAT becomes fully activated at 38°C. This is shown in panel (c), corresponding to a steady-state ACh concentration of 3.43±0.14 µM. No inhibitor of AChE or BuChE was used providing strong evidence that the ACh-synthesis by ChAT produces an ACh equilibrium level in the presence of naturally occurring amount of these ACh-hydrolyzing enzymes in CSF. **p<0.0013 compared to ACh level generated at the room temperature.

All of these ACh-related enzymes are simultaneously present in their active forms. Therefore, it is imperative to determine if ChAT is capable of maintaining certain levels of ACh in extracellular fluids, such as plasma and CSF, in the presence of fully functional degrading enzymes (**Fig. *3a***). In these experiments, we omitted eserine, i.e. the inhibitor of AChE or BuChE. Experiments were performed at two different temperatures, since AChE and BuChE are highly active at both room temperature and 38°C, while ChAT first becomes fully activated at 38°C [32] (**Fig. *3b–c***). We also included accessory enzymes and substrates to regenerate the required co-factor (acetyl-coenzyme-A) for the synthesis of ACh. At room temperature, the synthesized ACh concentration reached 2.11±0.27 µM within one hour (**Fig. *3b***). This was lower than the steady-state ACh concentration produced at 38°C, when ChAT became fully activated (**Fig. *3c***, 3.43±0.14, *p*<0.0013).

Experiments were also performed without adding choline to the samples, since endogenous choline concentration was sufficiently high (31±2 µM). A similar steady-state level of ACh was observed at both temperatures (data not shown).

### ChAT is Secreted by Human Embryonic Cells, Primary Human Astrocytes and Activated Mouse Lymphocytes

We next addressed whether or not ChAT is a secreted protein. This was tested first by using human embryonic stem (hES) cells, where the culture medium was assayed by sandwich ELISA. These cells secreted substantial amounts of ChAT into the medium (**Fig. 2c**). Next, we also quantified the amount of ChAT released by human-brain primary astrocytes into culture medium (**Fig. 2c**).

Lymphocytes also possess the essential components of the cholinergic system, including ChAT and both muscarinic and nicotinic ACh receptors [9], [13]. However, it is unclear how ACh is stored in the cytoplasm of these cells, since there is no evidence of expression of the vesicular ACh transporter (VAChT) [33]. This may indicate that lymphocytes synthesize ACh on demand and release it without storage [33]. A recent report has suggested that vagal-nerve activation stimulates a subpopulation of regulatory T-helper cells, which then migrate to the spleen (which has no direct vagal innervation) and release ACh to regulate the immune phenotype of spleen cells [34]. An alternative scenario is that these regulatory T-cells not only release ACh, but also active ChAT. In order to test this lymphocytes isolated from mice spleen were stimulated with activating CD3 antibodies or LPS.

**Figure 4:**
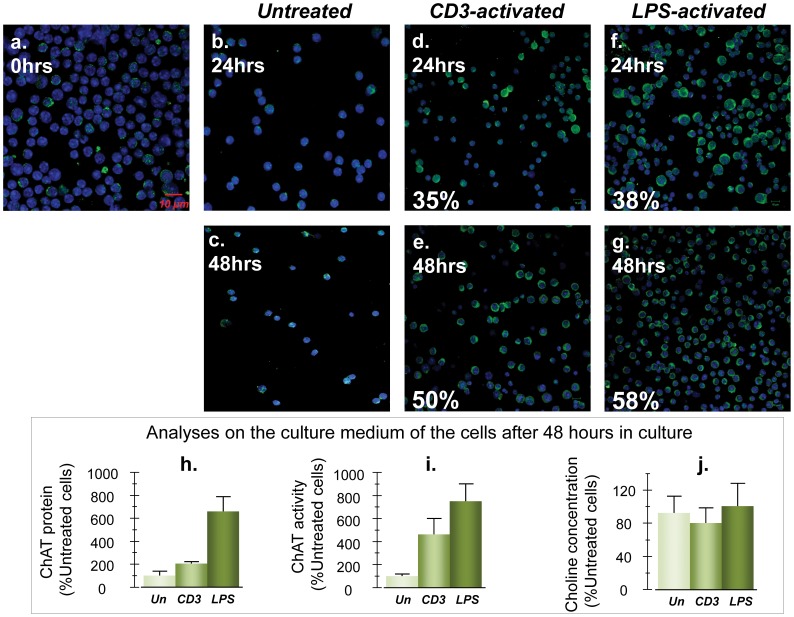
ChAT, rather than acetylcholine, is released by activated mouse lymphocytes. (a) Isolated mouse splenocytes that were directly mounted on microscope slide and examined for expression of ChAT. (b–c) The cells without stimulation, after being cultured for 24 and 48 hours. (d–e) The cells after being stimulated with anti-CD3 antibody. (f–g) The cells after being stimulated with LPS. Initially (a) or under resting conditions (b–c) very little ChAT immunoreactivity is detected in the cytoplasm of the splenocytes. In contrast, stimulation with CD3 antibody (d–e) and, in particular, with LPS (f–g) resulted in the expression of ChAT in the majority of the cells. (h) The measured ChAT protein that was released by the cells into the medium after 48 hours. (i) The corresponding analysis of ChAT activity in the medium. (j) The overall concentration of choline in the medium. A comparison between the measured levels of ChAT (h–i) and the choline concentration (j) in the culture medium reveals that the stimulated cells released ChAT rather than ACh into the medium, since the overall levels of choline in all three culture medium are essentially similar (j).

Very few cells displayed positive ChAT immunostaining under resting conditions, and no significant change occurred upon incubation for up to 48 hours (**Fig. 4a–c**). In contrast, both CD3 and (even more so) LPS stimulation resulted in a dramatic increase of ChAT cytoplasmic immunolabeling after 24 and 48 hours so that all cells essentially became ChAT immunopositive (**Fig. 4d–g**).

We next measured the activity and protein concentration of ChAT in the culture medium of these cells. After 48 hours in vitro, the relative levels of the ChAT protein displayed a two- and 6.5-fold increase in the cell medium of the CD3- and LPS-stimulated cells compared to the untreated cells, respectively (**Fig. 4h**). The corresponding ChAT activity was increased by about 4.5- and seven-fold in the cell medium of the CD3- and LPS-stimulated cells, respectively (**Fig. 4i**). In addition, we found no significant differences with regard to overall concentration of choline in the culture mediums (average 7.20±0.96 µM, **Fig. 4j**), which argues against direct release of only ACh into the medium by lymphocytes.

### Levels of ChAT in CSF Show a BCHE-K Allele Dose-dependency

**Figure 5:**
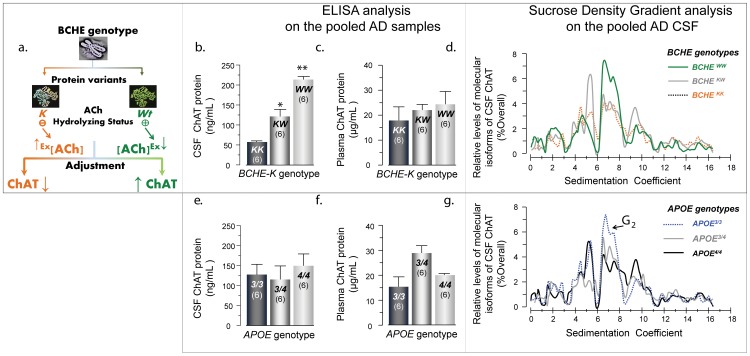
Extracellular levels of ChAT proteins in CSF are influenced by the BCHE-K genotype. (a) Schematic outline of our hypothesis that if the maintenance of extrasynaptic ACh equilibrium by extracellular ChAT are physiologically regulated, then ChAT levels should show a BCHE-K dosage-dependency. This is because the phenotypic display of BCHE gene shows a K allele dosage-dependency that is 30–50 percent lower than the wild-type carriers [35]. (b) Indeed, the protein levels of ChAT exhibit a strong BCHE-K dependent pattern in CSF. This pattern is less pronounced in plasma (c). This may reflect the absolute dominance of AChE activity in the blood circulation (see also the Fig. 2d) making the physiological regulation of ChAT activity in circulation less dependent on BuChE activity. (d) The independent sucrose-density gradient (SDG) analysis of ChAT levels with regards to for BCHE-K genotype. This confirms the observation in (b), and also suggests that a differential pattern of assembly of ChAT subunits may be involved. This is because the G_2_ ChAT molecular forms dominates in the CSF of wild-type carriers of BCHE (BCHE^W/W^, green line), while the monomeric ChAT forms (G_1_) is the most abundant molecular form in the heterozygotes (BCHE^K/W^, grey line) or K-homozygotes (BCHE^K/K^, orange line). (e–f) The impact of the risk allele of AD, APOE4 on the levels of ChAT protein in pooled CSF and plasma samples. (g) The SDG analysis of CSF ChAT for APOE genotypes. An APOE4-dependent pattern is most apparent in the levels of G_2_ ChAT form (compare the G_2_ peaks in g). This supports the above notion of differential pattern of assembly of ChAT subunits. The digits in the column bar represent the number of pooled samples that were analyzed.

The current investigation was initiated as a proof-of-concept of the physiological and pathological contribution of BAβACs in CSF of patients with AD. Since differential levels of ApoE and BuChE may affect the formation of BAβACs, it is relevant to test if CSF levels of ChAT are related to genetic AD risk factors such as APOE4 and BCHE-K. In particular, since functional variability in BuChE activity in CSF is strongly influenced by a gene-dosage effect of the number of BCHE-K alleles [35]. Thus, since ChAT in extracellular fluids is physiologically regulated, levels of ChAT in CSF may also be related to BCHE genotypes (**Fig. 5a**).

We compared ChAT activity and protein levels in nine different AD plasma and CSF samples, pooled on the basis of their combination of APOE4 and BCHE-K genotypes. The protein level of ChAT in CSF showed strong BCHE-K allele dose-dependency. The amount of ChAT protein was more than two- and four-fold less in the pooled CSF from the BCHE-K homozygous AD patients, compared respectively to heterozygotes and wild-type homozygotes (**Fig. 5b**). The corresponding analysis of the pooled plasma samples had similar findings, although the K allele gene dosage-effect dependency was less pronounced (**Fig. 5c**). This may be due to the absolute dominance of RBC AChE, rather than plasma BuChE, activity in the circulation (**Fig. 2d**).

Independent sucrose-density gradient analysis confirmed and expanded the above observations, with regard to differential levels of CSF ChAT protein (**Fig. 5d** vs. **5b**). The sedimentation analysis revealed that the difference was most likely related to the amount of the G_2_ variant of ChAT in BCHE-*Wt* homozygotes and the G_1_ and G_4_ variants in the heterozygotes compared to the BCHE-K homozygotes (compare the corresponding peaks in **Fig. 5d**).

We also compared the expression of ChAT in relation to APOE genotypes (**Fig. 5e–g**), as the ε4 allele of this gene is the main genetic risk factor for sporadic AD. No significant differences were observed neither with regard to the overall ChAT levels in pooled AD CSF (**Fig. 5e**) nor plasma (**Fig. 5f**). However, the sedimentation analysis indicated different molecular forms of ChAT in CSF, particularly the G_2_ variant, which was related to APOE4 allele dosage (**Fig. 5g**). The impact of APOE4 genotypes might have been masked when only overall ChAT protein was measured (**Fig. 5e–f**). This may indicate a differential arrangement of ChAT subunits in the heavier molecular forms of ChAT (**Fig. 5g** **)**.

### CSF ChAT is Upregulated in Autoimmune Neuroinflammation

Taken together, these observations indicate that higher ChAT protein is present in CSF and plasma samples with greater ACh hydrolyzing phenotype. This supports the notion that the levels of these interrelated enzymes are physiologically balanced in extracellular fluids. The fact that ACh exerts an immunomodulatory role suggests that it can play a role in conditions of neuroinflammation driven by an adaptive immune response. We therefore determined the levels of ACh- generating and degrading enzymes, respectively, in the CSF of patients with multiple sclerosis (MS) and a matching control group consisting of patients with non-inflammatory neurological/psychiatric conditions.

**Figure 6:**
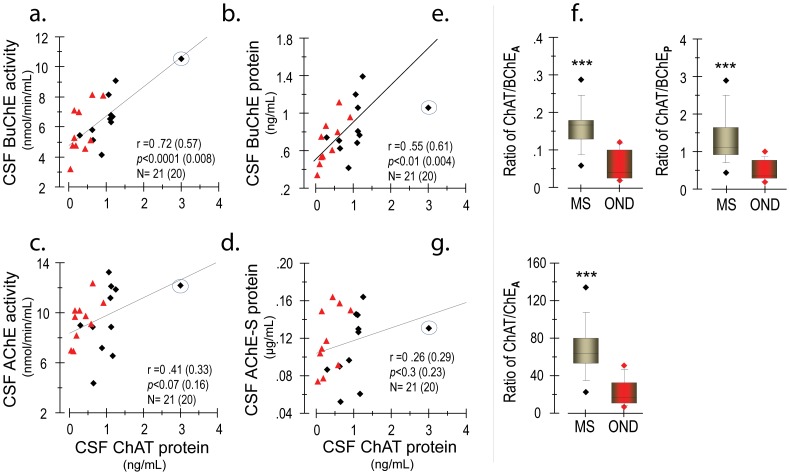
The extracellular protein expression of ChAT is strongly linked to the phenotypic display of BuChE, rather than the AChE level, in the CSF. Levels of ChAT protein show a high correlation with BuChE activity (a) and protein (b) in CSF. In contrast, the level of ChAT exhibits no significant correlation with levels of AChE activity (c) and the synaptic AChE-S protein (d) in the CSF. Panels (e–g) show the ratios of ChAT to the levels of the counteracting enzymes, which may serve as an index of extracellular ACh equilibrium. These ratios highly differ among patients with multiple sclerosis (MS) and patients with other neurological disorders (OND). These observations may suggest a physiological mechanism trying to rebalance the inflammatory processes by shifting the extracellular ACh equilibrium state, and thereby suppressing excessive inflammatory responses among MS patients. ***p<0.001. The digits in the parentheses show the statistics after exclusion of the outlier (as circled in a–d), indicating that this does not affect the correlation between BuChE and ChAT.

Consistent with the observation in AD, we found that CSF levels of BuChE, but not AChE (**Fig. 6a–b** vs **6c–d**), highly correlated to the corresponding levels of ChAT. Both BuChE and ChAT were elevated in MS patients compared to controls. These findings were closely in line with the observation that functional variability in BuChE may play an important role for the regulation of cholinoceptive astroglial activity in CNS and intrathecal levels of pro-inflammatory cytokines [36].

The ratio of ChAT to cholinesterases (ChAT/ChE-ratio) may be indicative of the extrasynaptic ACh equilibrium status, since a high ChAT/ChE ratio plausibly will lead to more generated than degraded ACh. This ratio was significantly higher in the CSF of patients with MS compared to patients with other non-inflammatory neurological conditions (OND) (**Fig. 6e–g**). This finding lends further support for the notion of ACh as an immune regulator, possibly as a counter-regulator in neuroinflammation caused by aberrant T-cell activation.

### CSF ChAT is Strongly Correlated to CSF Levels of Complement Components

**Figure 7:**
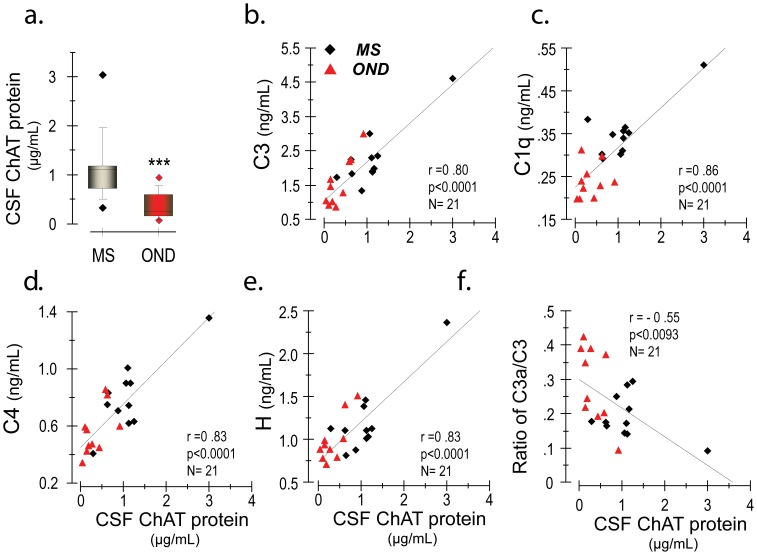
CSF levels of ChAT protein highly predict the levels of components of the innate immune complement system. Multiple sclerosis (MS) is an inflammatory neurodegenerative disease with potential involvement of the cholinergic anti-inflammatory pathway. (a) The ChAT protein level is more than two-fold higher in the CSF of MS patients compared to patients with other neurological disorders (OND). (b–f) There are high correlations between the CSF ChAT protein and CSF levels of the various components of the complement system, as well as with the ratio of C3a/C3. These findings support the notion of a role for ACh as an immune regulator in MS, and further suggest increased ChAT release as a possible counter regulatory mechanism in conditions of neuroinflammation caused by aberrant T-cell activation. ***p<0.001.

The complement system constitutes an important part of the innate immune system. Upstream complement components are expressed by cholinoceptive cells in the CNS, such as microglia and astrocytes. ACh equilibrium in the brain interstitial fluid may therefore influence expression of complement components. We have recently found that TNF-α mediated increase in expression of complement C3 in cultured rat, mice and human astrocytes is attenuated by addition of ACh to the culture medium (unpublished data and [36]). We hence determined the levels of several complement factors in the patients with MS and controls. We then compared this to ChAT levels. ChAT levels were more than two-fold higher in the CSF of patients with MS compared to the age-matched control group (**Fig. 7a**). Furthermore, CSF ChAT levels displayed a highly significant correlation to all of the measured components of the complement system (**Fig7. b–f**).

### Gene Expression and Release of ChAT by Astrocytes is Modulated by TNF-α

**Figure 8:**
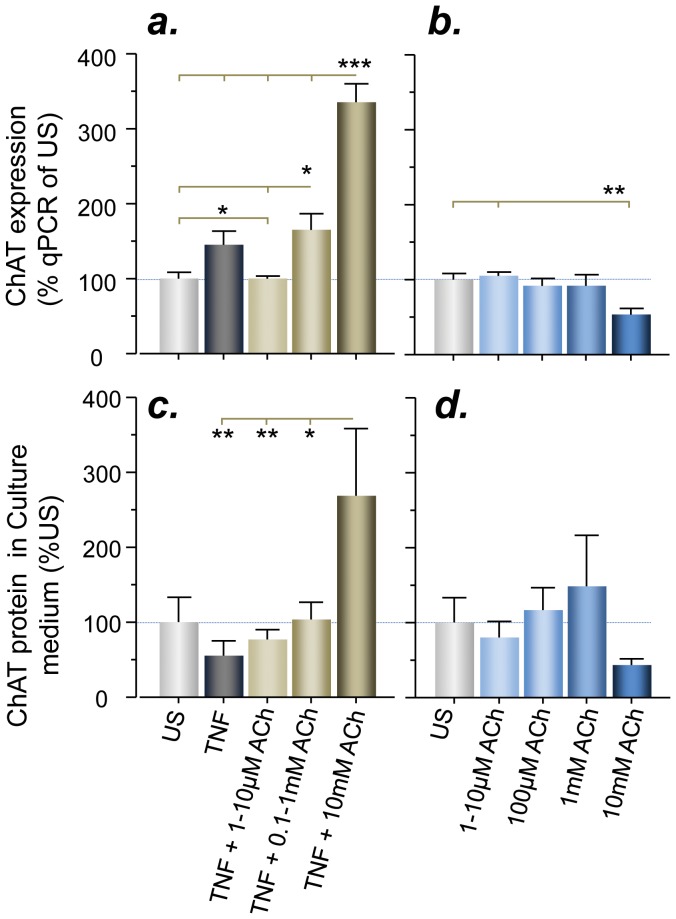
Regulatory changes in the ChAT level induced by TNF-α and acetylcholine in human primary astrocytes cell culture. (a) Stimulation with tumor necrosis factor α (TNF-α) alone, or in combination with micro- to milimolar concentration of ACh, affects the gene expression of ChAT in human-brain primary astrocytes. (b) ACh alone, at 10 mM concentration, reduces the expression of ChAT by ∼50 percent, contrasting the 3.5-fold increased ChAT expression with simultaneous stimulation with TNF-α. (c–d) The corresponding changes in the release of ChAT into the astrocyte culture medium. TNF-α combined with milimolar concentration of ACh resulted in a strong release of ChAT into the culture medium of the astrocytes (c) but ACh alone reduced the release of ChAT (d). *p<0.05, **p<0.01, ***p<0.001.

We then looked at whether treatment of human-brain primary astrocytes by TNF-α alone, or combined with various ACh concentrations, modulates the expression and release of ChAT. TNF-α, or combined with milimolar concentrations of ACh, elevated ChAT gene expression in astrocytes (**Fig. 8a**).

However, TNF-α increased the release of ChAT into the culture medium (**Fig. 8c**) when used with 10 mM of ACh. In contrast, ACh alone at up to 1 mM concentration did not change, but significantly reduced ChAT gene expression at 10 mM (**Fig. 8b & d**). These findings suggest that astrocytes are not only cholinoceptive, but may also act as cholinergic cells and actively release ChAT into interstitial fluid. Therefore, astrocytes may be one of the sources of ChAT in the extracellular fluids.

## Discussion

We demonstrated a number of findings that constitute compelling evidence that the acetylcholine-synthesizing enzyme, ChAT, is not only an intracellular enzyme present in the cytosol of cholinergic cells, but is also present in extracellular compartments. Thus, functionally intact ChAT is present at high levels in human CSF and plasma and can maintain steady-state equilibrium of extrasynaptic ACh in presence of the fully active degrading enzymes, AChE and BuChE. Furthermore, ChAT is readily secreted into the culture medium of both hES cells, activated lymphocytes and human-brain primary astrocytes. These findings call for a revision of the traditional assumption that ChAT is solely a cytosolic enzyme, mainly localized to cholinergic nerve terminals of the central, autonomous, and peripheral nervous systems.

Furthermore, we extended our previous findings regarding the presence of certain hybrid, molecular complexes in CSF of AD patients[4]–[6], [14], [35], which we have termed BAβACs [14].

**Figure 9:**
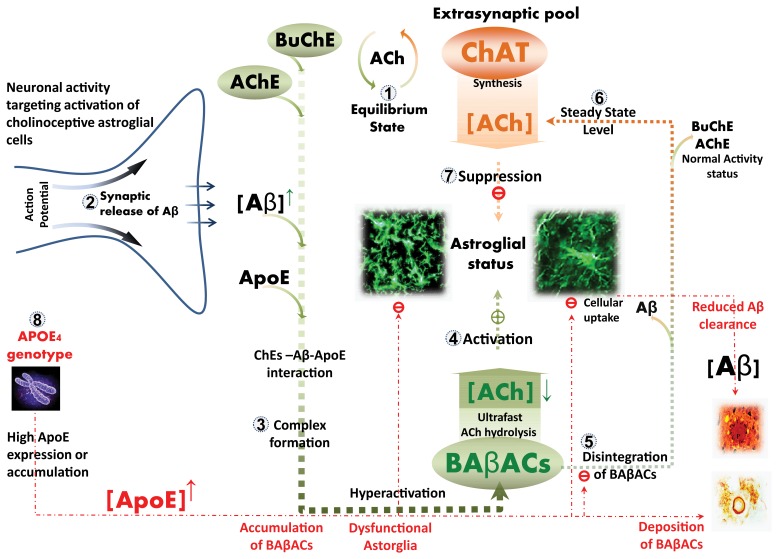
Hypothetical pathway for regulation of non-excitable cholinoceptive cells through distant action of extrasynaptic ACh. Extrasynaptic ACh can exert long distant regulatory effects on non-excitable cholinoceptive cells, such as microglia, astrocytes, oligodendrocytes and endothelia, mediated mainly through activation of nicotinic α7-ACh receptors [1]. However, extracellular ACh is highly labile due to extracellular presence of the ACh-degrading enzymes, AChE and BuChE. (1) The finding here of presence of extracellular ACh-synthesizing enzyme, ChAT, proposes a model in which continuous synthesis of ACh counteracted by breakdown could maintain a steady-state equilibrium, which can regulate glial activation status. (2) This equilibrium may also be influenced by neuronal activity, since action potentials by cholinergic neurons, for example, may lead to synchronized production and release of Aβ peptides into the interstitial fluid [7]. (3) These Aβ peptides can interact with AChE and BuChE, and temporarily form BAβA-complexes (BAβACs), in which these enzymes acquire ultrafast catalytic activity, plausibly through induction of certain conformational change in their tertiary structures [14]. (4) The hyperfunctional BAβACs will then effectively shift the equilibrium state towards lower ACh, which in turn leads to the activation of glia with expression of, for example, complement factors. (5) Reuptake of Aβ peptides will result in disintegration of BAβACs, (6) normalization of the steady-state balance, and (7) the initial astroglial status. (8) In AD the presence of the ε4 allele of apolipoprotein E (APOE) gene may disturb the balance additionally due to dysfunctional Aβ re-uptake (clearance) and/or high levels of ApoE protein, a common condition among APOE4 carriers [4]. High ApoE protein will prolong the interaction between cholinesterases and Aβ, leading to gradual accumulation and deposition of BAβACs in the brain. The end stage, BAβACs is deposited together with Aβ peptides in the brain as parts of the Aβ plaques and cerebral amyloid angiopathies, two hallmarks of Alzheimer’s disease brain, which also explains the documented presence of BuChE, AChE and ApoE in these Aβ deposits [65].

The physiological role of BAβACs has however been very perplexing, because it is difficult to conceive the possible functional impacts of hyper-activating two of the most abundant and intrinsically efficient ACh-degrading enzymes (i.e. AChE and BuChE). The relevance of this question has been increased by the relatively recent observation that ACh plays an important immune regulatory effect outside of neuronal synapses, where ACh is assumed to diffuse over longer distances in the presence of abundant ACh-degrading enzymes. The finding here that ChAT is abundant in extracellular compartments, such as CSF and plasma, makes it possible to reconcile this model with ACh’s short half-life (**Fig. 9**). It implies that a continuous regeneration of ACh counteracts the effect of degrading enzymes to maintain a steady-state, extrasynaptic ACh equilibrium. This is supported by the finding that functionally active ChAT is present in CSF and plasma, and can maintain ACh equilibrium in the presence of physiological levels of AChE and BuChE. This proposes a role for BAβACs in shifting this equilibrium, upon synaptic release of Aβ peptides [7].

We previously hypothesized that ApoE and Aβ attenuate cholinergic transmission through formation, gradual accumulation, and ultra-activation of BAβACs, in turn causing low-degree systemic inflammation in AD [5], [6]. Our findings are in line with this assumption and also indicate that ApoE exert its pathological role in AD by facilitating abnormal accumulation of BAβACs, and thereby disruption of proper neuronal and non-neuronal cholinergic signaling.

Another fundamentally important question concerns a native physiological function for Aβ peptides. It is difficult to conceive that such sophisticated and complex enzymatic machinery has been evolved for the universal production and release of Aβ peptides in the brain (which seems to be synchronized with synaptic activity [7]), without any physiological function. Our findings and other research suggest that Aβ may play a key role in regulating extracellular ACh, either through specific hierarchical phosphorylation-dependent increase of ChAT activity [37], [38] and/or activity-synchronized release of Aβ in interstitial fluid [7] (**Fig. 9**). In our model AChE and BuChE within the BAβACs act as the effector target of Aβ peptides [4], [14]. Interestingly, Aβ peptides also decreases the activity of ChAT [39]. This means that the ACh equilibrium might be affected by Aβ both at synthesis and degradation.

Non-neuronal cholinergic signaling are of special relevance, since approximately 90 percent of nonexcitable cells in the brain are cholinoceptive and highly sensitive to changes in extracellular ACh levels. This could explain the widespread metabolic and hemodynamic changes characterizing AD brain pathology. Thus the current findings highlight the necessity of further studies to provide more direct evidence for our hypothetical regulatory pathway of central cholinoceptive cells through the extra synaptic ChAT-Aβ-BAβAC axis (**Fig. 9**).

Our findings suggest that release of ChAT by cholinergic cells into extracellular fluid provides a mean for ACh to exert hormone-like activity on long-distance targets. This provides the essential missing link for the well-established cholinergic anti-inflammatory hypothesis [40], [41]. In this model, it is well-established that vagus-nerve stimulation during endotoxemia specifically attenuates TNF-α production by macrophages residing in the spleen. However, this effect has been difficult to explain as the spleen lacks direct vagal innervations, and splenic nerves do not express ChAT or vesicular ACh transporter [42]. Recently the presence of ACh-synthesizing, memory T-cells has been suggested to explain the inhibition of cytokine production by vagus-nerve stimulation in the spleen [34]. According to this, vagus-nerve stimulation activates memory T-cell populations, which then migrate to the spleen and regulate macrophage activation by release of ACh [34]. Our findings suggest these regulatory T-cells release ChAT rather than ACh, since anti-CD3 and particularly LPS activation caused a similar increase of both cytosolic (in agreement with [43]) and secreted ChAT protein, but the overall choline concentration in the culture medium did not change. This also provides an explanation for the observation that mRNA expression of VAChT is lacking in lymphocytes [44], which in turn suggests that lymphocytes are incapable of storing ACh in a similar manner to cholinergic neurons. It is also unlikely that direct release of ACh in the presence of abundant degrading enzymes could lead to such widespread effects. This is particularly true since the anti-inflammatory action of ACh is mediated through activation of α7-nAChRs, which are ligand-gated ion channels that require a continuous presence of ACh at sufficient concentrations to maintain activation. In contrast, with release of ChAT, a more long-lasting and widespread shift of steady-state equilibrium levels of ACh could be achieved [1], [2]. This is also in line with reported detectable levels of ACh in blood [9], [45], and with dynamic regulation of ChAT activity, but not its counteracting enzyme activity by hierarchical phosphorylation at several sites [38].

To the best of our knowledge, evidence for extracellular function of ChAT has only been reported in a study on human spermatozoa [24]. This report show that ChAT are localized outside the spermatozoa membrane and in the seminal fluids, whereby ACh is synthesized to act as a local hormone in coordinating contraction and relaxation cycles of spermatozoa flagella [24].

We also showed that one of the major sources of extracellular ChAT in the plasma might be lymphocytes, rather than the cholinergic neurons end terminals, since stimulation of spleenocytes resulted in a strong expression and a several-fold increased release of ChAT.

It is well-established that astrocytes and microglia are cholinoceptive cells, as they express nicotinic ACh receptors [46]. We showed here that astrocytes are actually cholinergic cells since they readily produced and released ChAT, both under resting and stimulatory conditions. Therefore, astrocytes may be one of the sources of ChAT in the brain.

Endothelial cells may be another source of the extracellular ChAT protein both in the peripheral and central compartments, since these cells have been shown to express ChAT and to produce ACh [8], [9]. There are also reports implicating endothelial cells in innate immunity and inflammation [47], [48].

We also found that astrocyte activation by TNF-α, in combination with milimolar concentrations of ACh, resulted in a several-fold increased release of ChAT. This may appear counterintuitive as we showed that stimulation of the astrocytes by high ACh alone resulted in decreased expression and release of ChAT. However, this may be explained by the enzymatic properties of ChAT. In contrast to AChE and BuChE, which can only catalyze breakdown of ACh, ChAT can perform both ACh breakdown and synthesis (see **Fig. *3a***).

One of the most apparent consequences of changes in the ACh equilibrium concerns the immunoregulatory function of cholinergic signaling through the action of ACh on cholinoceptive cell function involved in native and adoptive immune responses. We explored the possible clinical relevance of CSF ChAT levels by studying ChAT levels in the neuroinflammatory condition of MS. We found that CSF ChAT levels were significantly elevated in MS patients, compared to age-matched controls with non-inflammatory neurological conditions. CSF ChAT levels were closely correlated to levels of several complement factors. We also found a highly significant positive correlation between CSF ChAT levels and BuChE (but not AChE) activity or protein levels. As a relative index for the extracellular ACh equilibrium state, we calculated the ratio of ChAT to overall ChE activity in the CSF. A high ratio should indicate a low CSF ACh-hydrolyzing status and/or a high ACh-synthesizing status. Such a status would indicate a high extracellular ACh concentration. We found that this ratio to be two-fold higher in the CSF of the MS group than in the OND group. This observation suggests that increased ACh is a counter-regulatory response to inflammation, which may be driven by an aberrant T-cell response with autoimmune neuroinflammation. In the CNS compartment, immune cells are greatly outnumbered by CNS inherent neurons and glia. It is likely that a large proportion of the ChAT activity detected in the CSF stems from the latter. Regardless of the source, the findings of increased ChAT and BuChE indicate that this pathway is malfunctioning in patients with MS.

The current study also report an integrated simple, reliable and specific assay for simultaneous measurement of both activity and protein levels of ChAT in practically any biological fluid. We used undiluted or diluted human CSF and plasma, rather than concentrating the samples by centrifugation and filtration, which has been done in previous studies [27], [28], [30]. The lack of a simple ChAT assay and the noted handling of the samples are the most likely reasons why first now seventy years after its discovery we can show that ChAT exists extracellularly. Indeed, a careful search of preceding literatures ended up with five reports showing negligible extracellular ACh-synthesizing activity[21]–[25]. However, even these handful reports have been specifically disputed by just as many later reports[26]–[30]. This may also be due to other difficulties commonly encountered in measuring ChAT activity. These include the stability and/or solubility of the enzyme itself during purification procedures or perhaps due to an unidentified endogenous inhibitor of ChAT [49], [50], or a combination with methodological approaches [51].

The common view is that ChAT in the brain exists as a 68 kDa and a 80 kDa protein [32], [37]. We found multiple molecular forms of ChAT in plasma and CSF. Molecular weights ranged from 25 kDa to more than 300 kDa. In some previous studies, ChAT aggregates of 125 kDa or greater have been thought to be a result of purification artifacts, rather than a true molecular form of ChAT [32], [52]. However, the molecular forms found here are unlikely to represent such secondary aggregates of ChAT. They were readily separated by sucrose gradient analyses in nine different pooled CSF samples, all of which gave the same molecular pattern. This technique has been used for characterizing the molecular forms of the ACh-degrading enzymes, AChE, and BuChE that consist of various globular (G_n_) subunits. We here used the same nomenclature, where the monomeric G_1_ subunit of CSF ChAT had a Mw between 50–70 kDa, in close agreement with earlier reports [32], [52], and was the most abundant form in brain homogenates, as deduced by the reducing Western blot analysis. The G_2_ molecular form of ChAT had an Mw of 100–140 kDa, likely representing the assembly of two G_1_ ChAT subunits. It was the most abundant molecular complex of ChAT in CSF. The heavier G_4_ molecular form of CSF ChAT had an Mw ranging between 200–300 kDa, which fits well with assembly of four G_1_ subunits.

A much smaller ∼25 kDa subunit ChAT protein was detected in CSF, plasma and brain homogenates, both with immunoblotting and sucrose density gradient analysis. This may represent a partially degraded enzyme. However, reports indicate that the human gene for ChAT generates two alternatively spliced transcripts, which produce two protein variants of ChAT: a 68 kDa protein and a 27 kDa protein [20]. This is essentially identical to the 25 kDa subunit of ChAT shown here in the brain tissue homogenates, CSF and plasma. Reports suggest that this smaller subunit of ChAT lack catalytic activity but might regulate the activity of the full-length ChAT protein [20].

Interestingly, the level of CSF ChAT, in particular the G_2_ form, was associated with genetic risk factors of AD, APOE4 and BCHE-K genotypes [35], [53]–[57]. Recently, we have shown that functional variability in BuChE activity, due to either genetic polymorphism or high ApoE protein, regulates the intrathecal cytokine and astroglial biomarker profile in patients with AD [35], [36]. The findings here indicate that carriers of the wild-type BCHE have higher ChAT concentration in the plasma and CSF. BuChE activity itself also shows a strong BCHE-Wt gene-dosage dependency so that the wild-type homozygotes display 30–60 percent greater BuChE activity than carriers of the K allele [35]. Thus the differential levels of ChAT seem to reflect a physiological adaptation to the 30–60 percent higher ACh-hydrolyzing status of these subjects. This was supported by the comparative analysis between levels of these cholinergic enzymes in plasma and CSF. The much higher abundance of ChAT in plasma compared to CSF was shown to follow a similar biological balance in the levels of the counteracting ACh-degrading cholinesterases in the blood circulation relative to the CSF.

The findings reported here warrant further studies of the various molecular forms of ChAT not resolved here. For instance, do the subunits of ChAT assemble through disulfide bridges in a similar manner as the subunits of AChE and BuChE? Is there any membrane-anchored extracellular ChAT present on astrocytes or lymphocytes in similar fashion that the G2 AChE is anchored to the outer membrane of blood cells? Does the 25-kDa ChAT splice variant bind to the full-length ∼65 kDa variant, and has such a binding a regulatory role of the ChAT activity? There are also other critical issues such as the source of acetyl-Co enzyme A in extracellular fluids, which is required by ChAT for the synthesis of ACh. For instance, it is possible that this cofactor is released simultaneously by the cells together with ChAT.

## Materials and Methods

### Ethics Statement

All studies involving human subjects and animals had been approved by the Regional Ethical Review Board in Stockholm or the Research Ethics Committee of the South, Huddinge University Hospital. Written informed consent was obtained from each patient or the responsible caregivers prior to collection and storage of samples for research purposes in Biobank of the Karolinska University Hospital, Huddinge. This study was conducted according to the Declaration of Helsinki and subsequent revisions.

C57BL/6 mice were housed under standard conditions at the Department of Microbiology, Tumor and Cell Biology at both Karolinska Institutet and Karolinska University Hospital Huddinge, Stockholm, Sweden. All procedures were performed in conformance with both institutional and national guidelines.

### Pooled Samples of CSF, Plasma, and Postmortem Brain-tissue Homogenate

Cortical brain homogenates (BH) from parietal cortex of AD patients (n = 5, mean age 78±5 years, postmortem delay 26±2 hours, nonsmoking) and parietal cortex of non-AD controls (n = 5, mean age 82±2years, postmortem delay 21±4 hours, nonsmoking) were prepared as previously described [58]. The brain tissues were provided by the Netherland Brain Bank.

Nine pooled samples of CSF and plasma with defined APOE and BCHE-K genotypes were prepared by pooling equal volumes of at least three samples with identical genotypes. The pooled plasma and CSF samples were prepared from 179 patients with clinical diagnosis of mild-to-moderate AD, the selection criteria and the demographic data are the same as reported recently [35]. All samples had been collected prior to any treatment with cholinesterase inhibitors. The following nine genotype combinations of pooled samples were prepared: **A** (APOE^ε44^/BCHE*^WW^*); **B** (APOE^ε44^/BCHE*^KK^*); **C** (APOE^ε44^/BCHE*^WK^*); **D** (APOE^ε43^/BCHE*^WW^*); **E** (APOE^ε43^/BCHE*^KK^*); **F** (APOE^ε43^/BCHE*^WK^*); **G** (APOE^ε33^/BCHE*^WW^*); **H** (APOE^ε33^/BCHE*^KK^*); **I** (APOE^ε33^/BCHE*^WK^*) (**Fig. 1a**).

All pooled brain extracts and CSF/plasma samples were kept at −80°C until the assay.

### Dot-blot Analyses of ChAT

The pooled human CSF and plasma samples, as well as the pooled homogenates from the control and the AD brains, were used (undiluted and 2–64 fold diluted samples prepared in PBS, phosphate buffer saline; 50 mM, pH 7.4) and dot-blotted on nitrocellulose membranes (Hybond C, Amersham Life Science). The analysis was done according to standards procedures. The anti-ChAT antibodies were either a mouse monoclonal (MAB3447, 1/500, R&D System, UK) or a rabbit polyclonal antibody (PAB 14536, 1/2000; Abnova Corp. Taiwan). The corresponding HRP-conjugated secondary antibody was a goat anti-rabbit or a goat anti-mouse antibody (sc 2030 and sc2302, respectively; 1/2000, Santa Cruz Biotechnology). Finally, the membranes were incubated for five min in 3 mL/blot of ECL plus reagents (Amersham Life Science). The blots were then photographed with a digital camera (Fuji, LAS-3000), according to standard procedures.

### Immunoblotting Analyses of ChAT

Neat and diluted pooled human samples (CSF, plasma, and control and AD brain homogenates) were used. ChAT was detected by immunoblotting after resolution by sodium dodecyl sulfate polyacrylamide gel electrophoresis (SDS-PAGE) under reducing conditions, as described before [58]. The molecular mass (Mw) markers were Cruz Marker MW (sc-2035; Santa Cruz Biotechnology, CA) and prestained standard proteins (Cat no 161-0385, BioRad). The primary anti-ChAT (PAB14536) and the corresponding HRP-conjugated secondary antibodies were used to detect different bands of ChAT protein as described above. Each lane was loaded with 15 µL of a mixture, containing 10 µL of sample (diluted or undiluted,) and 5 µL of a 6x concentrated reducing Laemmli loading buffer.

### ChAT-specificity Analyses and Concentration Measurement using Sandwich ELISA

To ensure the identity of the proteins detected by the dot-blot and Western-blotting analyses, we designed sandwich ELISA assays using a combination of three different anti-ChAT antibodies and purified recombinant human ChAT protein (rhChAT, R&D systems) as a standard.

Briefly, the in-house constructed sandwich ELISA was set up as follows: High-binding plates (Nunc Maxisorb, Cat no 236366, Denmark) were coated with the anti-ChAT capturing antibodies [PAB14536 (1/2000) or MAB 3447 (1/250)], diluted in sodium carbonate buffer (pH 9.5). The plate was then blocked for two hours with the carbonate buffer, containing 5% w/v bovine serum albumin. All washing steps were done by 3 times of 5 min incubation with TBST^0.05%^ (10 mM Tris-HCl, pH 7.4; 0.9% NaCl; 0.05% v/v Tween-20).

All samples and standards were diluted in TBS buffer, containing 0.05% Triton-X100; 0.1% BSA and 1 mM EDTA. All samples or standards (recombinant human ChAT protein; rhChAT, R&D systems) were added in triplicate. The plate was incubated ON at 4°C under gentle orbital shaking. The plate was then incubated with one of the following detecting anti-ChAT antibodies: MAB 3447 (1/250), PAB 14536 (1/2000), or a biotin-labeled goat polyclonal anti-ChAT antibody (BAF3447; 1/500, R&D Systems) in TBST^0.05%^ buffer, containing 5% BSA and 0.01% NaN_3_. The reporter system was alkaline phosphatase (AP)-conjugated agent appropriate for the detecting antibody (AP-bovine anti-rabbit, sc 2376; AP-bovine anti-mouse, sc 2377 [both 1/2000, Santa Cruz Biotechnology]; or AP-streptavidin [1/2000, # 1093266, Roche] in ^3%^BSA-TBST^0.05%^ buffer).

Finally, the plate was incubated with the substrate solution (10 mM di-sodium *p-*Nitrophenyl phosphate, Cat no 73724, Sigma-Aldrich) in 1.0 M diethanolamine buffer, pH 9.8, containing 1 M MgCl, 0.01% NaN_3_. The reaction was monitored at 405 nm wavelength using an Infinite® M1000 Tecan microplate reader.

It should be mentioned here that the main scope of the current study was to demonstrate extracellular ChAT protein and activity. We therefore decided to use reagents recognizing both the various splice variants and the common and peripheral ChAT isoforms described in the “Introduction” section instead of reagents specific to a particular isoforms of ChAT.

### Sucrose-density Gradient Ultracentrifugation and Sedimentation Analysis

Molecular forms of ChAT were also separated by sucrose-gradient sedimentation technique and ultracentrifugation at 165000×g in a continuous sucrose gradient (5–20% w/v) for 18 hours at 4°C in a Beckman rotor (SW 41 Ti) as described before [58]. The sucrose gradients (10 mL/tube) were prepared in TBS (Tris-HCl, 10 mM, pH 7.4, containing 150 mM NaCl and 50 mM MgCl_2_). The pooled CSF (0.5 mL) or brain homogenate (0.3 mL) samples were applied on top of the gradients. Approximately 50 fractions were collected from the bottom of each tube. Concentration of ChAT protein in the collected fractions was then determined by sandwich ELISA as outlined above. Enzymes of known sedimentation coefficient, bovine liver catalase (11.4S; ∼250 kDa) and calf intestinal alkaline phosphatase (6.1S; ∼140 kDa), were used in the gradients to estimate the sedimentation coefficient of different molecular forms of ChAT.

### ChAT Activity Determination Assay

Neat or diluted human samples (CSF and plasma, as well as AD and control brain homogenates) were used for this assay. In part of the analysis, individual CSF (n = 10) and plasma (n = 10) samples were used instead of the pooled samples, described in the aforementioned section. These samples were also from patients with mild-to-moderate AD with the following demographic data [all were carriers of APOE4 allele, but non-carriers of the BCHE-K allele; the mean age (±S.E.M.) was 70±3 years, gender (7 Female/3 Male); average global cognition status was 23±1 (±S.E.M.), which was assessed by MMSE (mini-mental state examination) test]. All samples were collected prior to treatment with cholinesterase inhibitors. When diluted sample was used, the dilution buffer was TBS-Tr^0.05%^ (filtered TBS 10 mM pH 7.4, containing 1 mM EDTA and 0.05% Triton X100).

The ChAT assay presented here is in principle based on the chemiluminescence assay for ChAT described by Menzel et al.,1988 [59]. Several major modifications were introduced to improve the handling of samples and their control blanks, and the detection system as described below. Details of the final protocol of this ChAT activity assay and its validation will be described elsewhere. Briefly, high binding 384-well microtiter plates (Nunc Maxisorb, Denmark) were precoated ON at 4°C with 75 µL/well of the MAB 3447 (1/250 in the coating buffer). After blocking with 100 µL/well of BSA (5%w/v in the coating buffer), triplicates of the samples or the ChAT protein-calibrated pooled plasma standards (all 10 µL/well), and a series of choline chloride standards (50 µL/well), were applied into the wells of a microtiter plate. All samples and the ChAT-calibrated plasma standards were applied both as native (unmodified) samples and as denatured (by heating in a thermal cycler 3 times 8 minutes at 98°C, which inactivated ChAT). The denatured samples served as controls for both the ChAT activity and the endogenous concentration of choline in the samples.

Then 40 µL of ***Cocktail-A*** were added to each well. The ***Cocktail-A*** was prepared in the TBS-Tr^0.05%^ buffer and contained 62.5 µM of lithium salt of acetyl coenzyme-A (ACoA; A2181, Sigma-Aldrich); 1.25 U/ml of phosphotransacetylase (P2783, Sigma-Aldrich); 8.75 mM of lithium potassium acetyl-phosphate (#01409, Sigma-Aldrich); 6.25 µM choline chloride and 0.75 mM of eserine hemisulfate (E8625, Sigma-Aldrich ). The function of phosphotransacetylase and acetyl phosphate was to regenerate back the ACoA in the reaction wells [60]. The choline standards were prepared by two-fold serial dilution in the TBS, starting from 50 µM choline chloride, and 50 µL/well were applied in triplicate on the same microtiter plate. ***Cocktail-A*** was not added to the choline standards, which quantified the amount of choline remaining in the wells at the end of the ChAT reaction. The plate was then sealed and incubated in a humid chamber for one hour at 38°C, under constant, gentle linear shaking. Immediately, after the incubation, 25 µl/well of **Cocktail B** (50 mM PBS, pH-7.6, containing 0.93 U/ml of choline oxidase [C5896, Sigma-Aldrich]; 1/5000 Streptavidin-HRP [#43-4323, Invitrogen]; 3.0 mM 4-aminoantipyrine [A4382, Sigma-Aldrich] and 6.3 mM phenol [P3653, Sigma-Aldrich]) was added to all the wells, including the choline standards. Reaction absorbance was then monitored using a microplate spectrophotometer reader (Infinite® M1000, Tecan) at 500 nm wavelength.

ChAT activity (nmol/min/mL samples) was calculated according to the following formula: *ChAT activity
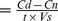
*, in which *Cd* and *Cn* are the measured choline concentrations (in *p*mol) in the denatured and native samples, respectively, *t* is the incubation time (in minutes) at 38°C, and *Vs* is the volume (mL) of the samples.

This should be noted that at the end of this step the plate can be sealed and incubated ON at 4°C. Then the plate is washed as before, and incubated with the detecting antibody, PAB 14536 (1/2000) and the subsequent steps in the sandwich ELISA described in the previous section. This procedure will with obvious advantages integrate the ChAT protein determination to the ChAT activity assay.

### MS and Control CSF Study Analyses

CSF samples from 10 patients with multiple sclerosis (MS) and 11 patients with other non-inflammatory neurological conditions (OND) were used. The MS and OND CSF samples were obtained from patients attending the Neurology Clinic, Karolinska University Hospital, Solna. Clinical examinations were performed by specialists in neurology, and all MS patients fulfilled the McDonald criteria [61]. All MS patients were evaluated at time of sampling with the Expanded Disability Status Scale (EDSS) [62] by a certified rater.

The OND group consisted of patients with non-inflammatory neurological/psychiatric conditions with normal MRI scans and without signs of inflammatory activity in CSF in terms of pleocytosis or intrathecal IgG production. There were no significant concomitant diseases, such as infections. Corticosteroids had not been given within three months of sampling.

The CSF levels of C3, C3a, C1q, C4, and H components of the complement system were measured using a sandwich ELISA, essentially as previously described [63].

AChE and BuChE activities were determined as described [14]. Their respective protein levels were quantified using a custom-made ELISA, as previously described [31]. ChAT activity and protein levels were measured in the samples as outlined above. All quantifications were performed in a blinded fashion and patient allocation to the groups was performed before analysis by a single rater.

### Immunofluorescence and Confocal Microscopy

Spleen cells (10^6^ cells/mL), which were from C57BL/6 mice, were stimulated with lipopolysaccharides (LPS, 25 µg/mL) or with anti-CD3 (clone 2C11 at 5 µg/mL). The cell cultures were run in parallel in duplicates in RPMI1640 (HyClone Cat no SH3009601), supplemented with 10% fetal calf serum, 2 mM L-glutamine, 50 U/ml penicillin, 50 g/ml streptomycin, 10 mM HEPES, 1 mM sodium pyruvate and 50 µM 2-mercaptoethanol.

The cells and medium were collected after 24 and 48 hours for analysis. The cells were washed 3 times with PBS, and mounted on adhesion slides (ER-202W-AD-CE24, Thermo Scientific). The cells were fixed and prepared for the subsequent confocal microscopy as described [64], with minor modification. The cells were permeabilized with 0.2% saponin, instead of Triton-X100, for 15 min each. A rabbit polyclonal anti-ChAT antibody (AB143, Millipore, 1∶100) was used for the immunocytochemistry of ChAT in the mice lymphocytes.

### Human Astrocyte Cultures

Primary human astrocytes were obtained from ScienCell Research Laboratories (Cat no 1800). Cells were cultured in astrocyte medium (Sciencell Cat no 1801) in poly-L-lysine-coated T-175 flasks, and handled according to manufacturer’s protocols. Confluent cells were then detached, and seeded in a 48-well plate (3×10^5^ cells/well), and left in medium overnight to recover. Following this, the cells were stimulated with ACh or tumour necrosis factor-α (TNF-α) alone, ACh+TNF-α, or left unstimulated as control. ACh concentration varied between 1 µM to 10 mM and TNF-α concentration was kept constant at 20 ng/ml. The astrocytes were stimulated for 24 hours, after which the cells were lysed for RNA extraction and subsequent reverse transcriptase-PCR expressional analysis. The cultured mediums were saved for measuring AChE and BuChE activity and protein levels. All conditions were in triplicate. The pH of the medium was lowered to 7.1 to increase the stability of ACh. Recombinant human TNF-α was obtained from R&D systems (Cat no 210-TA-010). Acetylcholine chloride was obtained from Sigma (Cat no. A2661).

### RNA Preparation and RT-PCR

Cells were lysed in RLT buffer (Qiagen, Hilden, Germany) for total RNA preparation. Total RNA was extracted and purified. Column DNase I was treated using an RNeasy Mini kit (Qiagen) and RNase-Free DNase Set (Qiagen), according to the manufacturer’s protocols. The RNA was synthesized to cDNA by incubation with 5x iScript reaction mix (Bio-Rad) for five min at 25°C, 30 min at 42°C, and five min at 85°C. All steps were performed under RNase-free conditions. Real-time PCR was conducted using a three-step PCR protocol and Bio-Rad CFX manager software (version 2.1, Bio-Rad, Hercules CA). Samples were run in triplicate. All primers and probes were designed with primer blast (www.ncbi.nlm.nih.gov/tools/primer-blast), and checked for specificity with melt-curve analysis. The housekeeping gene glyceraldehyde 3-phosphate dehydrogenase (GAPDH) was used to normalize the mRNA expression levels of the studied transcripts. Normalized expression levels were calculated with Bio-Rad CFX manager.

The primers sequences were as follows: ***GAPDH:*** Forward: 5′-AGGGCTGCT TTTAACTCTGGTAAA-3′; Reverse: 5′-CATATTGGAACATGTAAACCATGTAGT TG-3′. ***CHAT:*** Forward: 5′-ATGGGCTCTTCTCCTCCTAC-3′Reverse: 5′-GTGGAGTCTTTCACGAGGAC-3′.

## References

[1] Parrish WR, Rosas-Ballina M, Gallowitsch-Puerta M, Ochani M, Ochani K, et al (2008) Modulation of TNF release by choline requires alpha7 subunit nicotinic acetylcholine receptor-mediated signaling. Mol Med 14: 567–574. 18584048 10.2119/2008-00079.Parrish PMC2435495

[2] Pavlov VA, Parrish WR, Rosas-Ballina M, Ochani M, Puerta M, et al (2009) Brain acetylcholinesterase activity controls systemic cytokine levels through the cholinergic anti-inflammatory pathway. Brain Behav Immun 23: 41–45. 18639629 10.1016/j.bbi.2008.06.011 PMC4533839

[3] van Westerloo DJ, Giebelen IA, Florquin S, Daalhuisen J, Bruno MJ, et al (2005) The cholinergic anti-inflammatory pathway regulates the host response during septic peritonitis. J Infect Dis 191: 2138–2148. 15898001 10.1086/430323

[4] Darreh-Shori T, Modiri N, Blennow K, Baza S, Kamil C, et al (2011) The apolipoprotein E epsilon4 allele plays pathological roles in AD through high protein expression and interaction with butyrylcholinesterase. Neurobiol Aging 32: 1236–1248. 19713000 10.1016/j.neurobiolaging.2009.07.015

[5] Darreh-Shori T, Modiri N, Lahaut P, Nordberg A (2009) Apolipoprotein E and Butyrylcholinesterase synergistically promote Ab peptides oligomerization. Alzheimer’s & Dementia. Available: http://www.sciencedirect.com/science/article/pii/S1552526009001915 5: P225 Accessed 2013 May 14.

[6] Darreh-Shori T, Modiri N, Nordberg A (2009) ApoE and amyloid beta deflate the cholinergic neurotransmission by boosting the activity and stability of cholinesterases in the brain. Alzheimer’s & Dementia; Available: http://www.sciencedirect.com/science/article/pii/S1552526009005858 5: P305 Accessed 2013 May 14.

[7] Cirrito JR, Yamada KA, Finn MB, Sloviter RS, Bales KR, et al (2005) Synaptic activity regulates interstitial fluid amyloid-beta levels in vivo. Neuron 48: 913–922. 16364896 10.1016/j.neuron.2005.10.028

[8] Wessler I, Kirkpatrick CJ (2008) Acetylcholine beyond neurons: the non-neuronal cholinergic system in humans. Br J Pharmacol 154: 1558–1571. 18500366 10.1038/bjp.2008.185 PMC2518461

[9] Kawashima K, Fujii T (2008) Basic and clinical aspects of non-neuronal acetylcholine: overview of non-neuronal cholinergic systems and their biological significance. J Pharmacol Sci 106: 167–173. 18285657 10.1254/jphs.fm0070073

[10] Serobyan N, Jagannathan S, Orlovskaya I, Schraufstatter I, Skok M, et al (2007) The cholinergic system is involved in regulation of the development of the hematopoietic system. Life Sci 80: 2352–2360. 17512954 10.1016/j.lfs.2007.04.017 PMC2873871

[11] Kurzen H, Wessler I, Kirkpatrick CJ, Kawashima K, Grando SA (2007) The non-neuronal cholinergic system of human skin. Horm Metab Res 39: 125–135. 17326008 10.1055/s-2007-961816

[12] Grando SA (2006) Cholinergic control of epidermal cohesion. Exp Dermatol 15: 265–282. 16512874 10.1111/j.0906-6705.2006.00410.x

[13] Grando SA, Kawashima K, Wessler I (2003) Introduction: the non-neuronal cholinergic system in humans. Life Sci 72: 2009–2012. 12628450 10.1016/s0024-3205(03)00063-8

[14] Darreh-Shori T, Forsberg A, Modiri N, Andreasen N, Blennow K, et al.. (2011) Differential levels of apolipoprotein E and butyrylcholinesterase show strong association with pathological signs of Alzheimer’s disease in the brain in vivo. Neurobiol Aging 32: 2320 e2315–2332. 10.1016/j.neurobiolaging.2010.04.028 20538374

[15] Veerhuis R (2011) Histological and direct evidence for the role of complement in the neuroinflammation of AD. Curr Alzheimer Res 8: 34–58. 21143154 10.2174/156720511794604589

[16] Wang Y, Hancock AM, Bradner J, Chung KA, Quinn JF, et al (2011) Complement 3 and factor h in human cerebrospinal fluid in Parkinson’s disease, Alzheimer’s disease, and multiple-system atrophy. Am J Pathol 178: 1509–1516. 21435440 10.1016/j.ajpath.2011.01.006 PMC3078443

[17] Anglade P, Larabi-Godinot Y (2010) Historical landmarks in the histochemistry of the cholinergic synapse: Perspectives for future researches. Biomed Res 31: 1–12. 20203414 10.2220/biomedres.31.1

[18] Oda Y (1999) Choline acetyltransferase: the structure, distribution and pathologic changes in the central nervous system. Pathol Int 49: 921–937. 10594838 10.1046/j.1440-1827.1999.00977.x

[19] Tooyama I, Kimura H (2000) A protein encoded by an alternative splice variant of choline acetyltransferase mRNA is localized preferentially in peripheral nerve cells and fibers. J Chem Neuroanat 17: 217–226. 10697248 10.1016/s0891-0618(99)00043-5

[20] Grosman DD, Lorenzi MV, Trinidad AC, Strauss WL (1995) The human choline acetyltransferase gene encodes two proteins. J Neurochem 65: 484–491. 7616201 10.1046/j.1471-4159.1995.65020484.x

[21] Johnson S, Domino EF (1971) Cholinergic enzymatic activity of cerebrospinal fluid of patients with various neurologic diseases. Clin Chim Acta 35: 421–428. 5125328 10.1016/0009-8981(71)90216-6

[22] Massarelli R, Froissart C, Mandel P (1977) Diurnal oscillation of choline acetyltransferase activity in human blood. Neurosci Lett 5: 95–101. 19604977 10.1016/0304-3940(77)90171-9

[23] Froissart C, Mandel P, Massarelli R (1979) Diurnal oscillation of choline acetyltransferase activity in human blood cells and higher enzymatic activity during pregnancy. Cell Mol Biol Incl Cyto Enzymol 24: 199–204. 574060

[24] Sastry BV, Janson VE, Chaturvedi AK (1981) Inhibition of human sperm motility by inhibitors of choline acetyltransferase. J Pharmacol Exp Ther 216: 378–384. 7463354

[25] Ibanez CF, Pelto-Huikko M, Soder O, Ritzen EM, Hersh LB, et al (1991) Expression of choline acetyltransferase mRNA in spermatogenic cells results in an accumulation of the enzyme in the postacrosomal region of mature spermatozoa. Proc Natl Acad Sci U S A 88: 3676–3680. 2023918 10.1073/pnas.88.9.3676 PMC51515

[26] Aquilonius SM, Eckernas SA (1976) Choline acetyltransferase in human cerebrospinal fluid: non-enzymatically and enzymatically catalysed acetylcholine synthesis. J Neurochem 27: 317–318. 956842 10.1111/j.1471-4159.1976.tb01588.x

[27] Eckernas SA, Maepea O, Sahlstrom L, Aquilonius SM (1979) Choline acetyltransferase activity in human blood and its diurnal oscillation–an artifact? Med Biol 57: 36–38. 35718

[28] Goodman DR, Adatsi FK, Harbison RD (1984) Evidence for the extreme overestimation of choline acetyltransferase in human sperm, human seminal plasma and rat heart: a case of mistaking carnitine acetyltransferase for choline acetyltransferase. Chem Biol Interact 49: 39–53. 6722939 10.1016/0009-2797(84)90051-6

[29] Mader M, Dickmann U (1988) A reevaluation of choline acetyltransferase activity in human cerebrospinal fluid and serum. J Clin Chem Clin Biochem 26: 857–861. 3069951 10.1515/cclm.1988.26.12.857

[30] DeKosky ST, Scheff SW, Hackney CG (1989) Acetylcholine synthesis in human CSF: implications for study of central cholinergic metabolism. Neurochem Res 14: 191–196. 2725819 10.1007/BF00969638

[31] Darreh-Shori T, Brimijoin S, Kadir A, Almkvist O, Nordberg A (2006) Differential CSF butyrylcholinesterase levels in Alzheimer’s disease patients with the ApoE epsilon4 allele, in relation to cognitive function and cerebral glucose metabolism. Neurobiol Dis 24: 326–333. 16973370 10.1016/j.nbd.2006.07.013

[32] Peng JH, McGeer PL, Kimura H, Sung SC, McGeer EG (1980) Purification and immunochemical properties of choline acetyltransferase from human brain. Neurochem Res 5: 943–962. 7207697 10.1007/BF00966135

[33] Fujii T, Takada-Takatori Y, Kawashima K (2008) Basic and clinical aspects of non-neuronal acetylcholine: expression of an independent, non-neuronal cholinergic system in lymphocytes and its clinical significance in immunotherapy. J Pharmacol Sci 106: 186–192. 18285654 10.1254/jphs.fm0070109

[34] Rosas-Ballina M, Olofsson PS, Ochani M, Valdes-Ferrer SI, Levine YA, et al (2011) Acetylcholine-synthesizing T cells relay neural signals in a vagus nerve circuit. Science 334: 98–101. 21921156 10.1126/science.1209985 PMC4548937

[35] Darreh-Shori T, Siawesh M, Mousavi M, Andreasen N, Nordberg A (2012) Apolipoprotein epsilon4 Modulates Phenotype of Butyrylcholinesterase in CSF of Patients with Alzheimer’s Disease. J Alzheimers Dis 28: 443–458. 22012848 10.3233/JAD-2011-111088

[36] Darreh-Shori T, Vijayaraghavan S, Lindblom PF R, Piehl F, Aeinehband S, et al.. (2013) Functional variability in butyrylcholinesterase activity regulates the intrathecal cytokine and astroglial biomarker profile in patients with Alzheimer’s disease. Neurobiol Aging, Inpress. 10.1016/j.neurobiolaging.2013.04.027 23759148

[37] Dobransky T, Brewer D, Lajoie G, Rylett RJ (2003) Phosphorylation of 69-kDa choline acetyltransferase at threonine 456 in response to amyloid-beta peptide 1–42. J Biol Chem 278: 5883–5893. 12486117 10.1074/jbc.M212080200

[38] Dobransky T, Rylett RJ (2005) A model for dynamic regulation of choline acetyltransferase by phosphorylation. J Neurochem 95: 305–313. 16135099 10.1111/j.1471-4159.2005.03367.x

[39] Nunes-Tavares N, Santos LE, Stutz B, Brito-Moreira J, Klein WL, et al (2012) Inhibition of Choline Acetyltransferase as a Mechanism for Cholinergic Dysfunction Induced by Amyloid-beta Peptide Oligomers. J Biol Chem 287: 19377–19385. 22505713 10.1074/jbc.M111.321448 PMC3365976

[40] Pavlov VA, Tracey KJ (2004) Neural regulators of innate immune responses and inflammation. Cell Mol Life Sci 61: 2322–2331. 15378203 10.1007/s00018-004-4102-3 PMC11138906

[41] Fujimoto K, Matsui M, Fujii T, Kawashima K (2001) Decreased acetylcholine content and choline acetyltransferase mRNA expression in circulating mononuclear leukocytes and lymphoid organs of the spontaneously hypertensive rat. Life Sci 69: 1629–1638. 11589503 10.1016/s0024-3205(01)01237-1

[42] Rosas-Ballina M, Ochani M, Parrish WR, Ochani K, Harris YT, et al (2008) Splenic nerve is required for cholinergic antiinflammatory pathway control of TNF in endotoxemia. Proc Natl Acad Sci U S A 105: 11008–11013. 18669662 10.1073/pnas.0803237105 PMC2504833

[43] Ni L, Acevedo G, Muralidharan B, Padala N, To J, et al (2007) Toll-like receptor ligands and CD154 stimulate microglia to produce a factor(s) that promotes excess cholinergic differentiation in the developing rat basal forebrain: implications for neurodevelopmental disorders. Pediatr Res 61: 15–20. 17211134 10.1203/01.pdr.0000249981.70618.18

[44] Fujii T, Yamada S, Watanabe Y, Misawa H, Tajima S, et al (1998) Induction of choline acetyltransferase mRNA in human mononuclear leukocytes stimulated by phytohemagglutinin, a T-cell activator. J Neuroimmunol 82: 101–107. 9526852 10.1016/S0165-5728(97)00195-1

[45] Fujii T, Yamada S, Yamaguchi N, Fujimoto K, Suzuki T, et al (1995) Species differences in the concentration of acetylcholine, a neurotransmitter, in whole blood and plasma. Neurosci Lett 201: 207–210. 8786841 10.1016/0304-3940(95)12180-3

[46] Yu WF, Guan ZZ, Bogdanovic N, Nordberg A (2005) High selective expression of alpha7 nicotinic receptors on astrocytes in the brains of patients with sporadic Alzheimer’s disease and patients carrying Swedish APP 670/671 mutation: a possible association with neuritic plaques. Exp Neurol 192: 215–225. 15698636 10.1016/j.expneurol.2004.12.015

[47] Saeed RW, Varma S, Peng-Nemeroff T, Sherry B, Balakhaneh D, et al (2005) Cholinergic stimulation blocks endothelial cell activation and leukocyte recruitment during inflammation. J Exp Med 201: 1113–1123. 15809354 10.1084/jem.20040463 PMC2213139

[48] Foldes G, Liu A, Badiger R, Paul-Clark M, Moreno L, et al (2010) Innate immunity in human embryonic stem cells: comparison with adult human endothelial cells. PLoS One 5: e10501. 20463927 10.1371/journal.pone.0010501 PMC2864770

[49] Singh I, Xu C, Pettegrew JW, Kanfer JN (1994) Endogenous inhibitors of human choline acetyltransferase present in Alzheimer’s brain: preliminary observation. Neurobiol Aging 15: 643–649. 7824057 10.1016/0197-4580(94)00059-x

[50] Andriamampandry C, Kanfer JN (1993) Inhibition of cytosolic human forebrain choline acetyltransferase activity by phospho-L-serine: a phosphomonoester that accumulates during early stages of Alzheimer’s disease. Neurobiol Aging 14: 367–372. 8367018 10.1016/0197-4580(93)90123-s

[51] Chao LP, Wolfgram F (1974) Activation, inhibition and aggregation of choline acetyltransferase (EC 2-3-1-6). J Neurochem 23: 697–701. 4473518 10.1111/j.1471-4159.1974.tb04393.x

[52] Singh VK, McGeer EG, McGeer PL (1975) Two immunologically different choline acetyltransferases in human neostriatum. Brain Res 96: 187–191. 1175001 10.1016/0006-8993(75)90595-8

[53] Ferris S, Nordberg A, Soininen H, Darreh-Shori T, Lane R (2009) Progression from mild cognitive impairment to Alzheimer’s disease: effects of sex, butyrylcholinesterase genotype, and rivastigmine treatment. Pharmacogenet Genomics 19: 635–646. 19617863 10.1097/FPC.0b013e32832f8c17 PMC4114757

[54] Lane R, Feldman HH, Meyer J, He Y, Ferris SH, et al (2008) Synergistic effect of apolipoprotein E epsilon4 and butyrylcholinesterase K-variant on progression from mild cognitive impairment to Alzheimer’s disease. Pharmacogenet Genomics 18: 289–298. 18334913 10.1097/FPC.0b013e3282f63f29

[55] Lehmann DJ, Johnston C, Smith AD (1997) Synergy between the genes for butyrylcholinesterase K variant and apolipoprotein E4 in late-onset confirmed Alzheimer’s disease. Hum Mol Genet 6: 1933–1936. 9302273 10.1093/hmg/6.11.1933

[56] Lehmann DJ, Nagy Z, Litchfield S, Borja MC, Smith AD (2000) Association of butyrylcholinesterase K variant with cholinesterase-positive neuritic plaques in the temporal cortex in late-onset Alzheimer’s disease. Hum Genet 106: 447–452. 10830913 10.1007/s004390000277

[57] Wiebusch H, Poirier J, Sevigny P, Schappert K (1999) Further evidence for a synergistic association between APOE epsilon4 and BCHE-K in confirmed Alzheimer’s disease. Hum Genet 104: 158–163. 10190327 10.1007/s004390050929

[58] Darreh-Shori T, Hellstrom-Lindahl E, Flores-Flores C, Guan ZZ, Soreq H, et al (2004) Long-lasting acetylcholinesterase splice variations in anticholinesterase-treated Alzheimer’s disease patients. J Neurochem 88: 1102–1113. 15009666 10.1046/j.1471-4159.2003.02230.x

[59] Menzel EJ, Walzer LR, Meissl G, Millesi H (1988) Chemiluminescence assay for choline acetyltransferase in tissue extracts by using immune adsorption on monoclonal antibody. Analytica Chimica Acta 205: 183–193.

[60] Nachmansohn D, Wilson IB (1955) [104] Choline acetylase. Methods Enzymol: Academic Press. 619–624.

[61] McDonald WI, Compston A, Edan G, Goodkin D, Hartung HP, et al (2001) Recommended diagnostic criteria for multiple sclerosis: guidelines from the International Panel on the diagnosis of multiple sclerosis. Annals of neurology 50: 121–127. 11456302 10.1002/ana.1032

[62] Kurtzke JF (1983) Rating neurologic impairment in multiple sclerosis: an expanded disability status scale (EDSS). Neurology 33: 1444–1452. 6685237 10.1212/wnl.33.11.1444

[63] Henningsson AJ, Ernerudh J, Sandholm K, Carlsson SA, Granlund H, et al (2007) Complement activation in Lyme neuroborreliosis–increased levels of C1q and C3a in cerebrospinal fluid indicate complement activation in the CNS. J Neuroimmunol 183: 200–207. 17157926 10.1016/j.jneuroim.2006.10.022

[64] Behbahani H, Pavlov PF, Wiehager B, Nishimura T, Winblad B, et al (2010) Association of Omi/HtrA2 with gamma-secretase in mitochondria. Neurochem Int 57: 668–675. 20705111 10.1016/j.neuint.2010.08.004

[65] Mesulam MM, Geula C (1994) Butyrylcholinesterase reactivity differentiates the amyloid plaques of aging from those of dementia. Ann Neurol 36: 722–727. 7979218 10.1002/ana.410360506

